# Drive for consumption, craving, and connectivity in the visual cortex during the imagery of desired food

**DOI:** 10.3389/fnagi.2013.00077

**Published:** 2013-11-27

**Authors:** Jessica Bullins, Paul J. Laurienti, Ashley R. Morgan, James Norris, Brielle M. Paolini, W. Jack Rejeski

**Affiliations:** ^1^Department of Radiology, Wake Forest University School of MedicineWinston-Salem, NC, USA; ^2^Translational Science Center, Wake Forest UniversityWinston-Salem, NC, USA; ^3^Department of Mathematics, Wake Forest UniversityWinston-Salem, NC, USA; ^4^Department of Health and Exercise Science, Wake Forest UniversityWinston-Salem, NC, USA; ^5^Department of Geriatric Medicine, Wake Forest UniversityWinston-Salem, NC, USA

**Keywords:** food craving, network science, visual cortex, power of food scale, older adults

## Abstract

There is considerable interest in understanding food cravings given the obesogenic environment of Western Society. In this paper we examine how the imagery of palatable foods affects cravings and functional connectivity in the visual cortex for people who differ on the power of food scale (PFS). Fourteen older, overweight/obese adults came to our laboratory on two different occasions. Both times they ate a controlled breakfast meal and then were restricted from eating for 2.5 h prior to scanning. On 1 day they consumed a BOOST^®^ liquid meal after the period of food restriction, whereas on the other day they only consumed water (NO BOOST^®^ condition). After these manipulations, they had an fMRI scan in which they were asked to image both neutral objects and their favorite snack foods; they also completed visual analog scales for craving, hunger, and the vividness of the imagery experiences. Irrespective of the BOOST^®^ manipulation, we observed marked increases in food cravings when older, overweight/obese adults created images of favorite foods in their minds as opposed to creating an image of neutral objects; however, the increase in food craving following the imagery of desired food was more pronounced among those scoring high than low on the PFS. Furthermore, local efficiency within the visual cortex when imaging desired food was higher for those scoring high as compared to low on the PFS. The active imagery of desired foods seemed to have overpowered the BOOST^®^ manipulation when evaluating connectivity in the visual cortex.

## INTRODUCTION

The increase in obesity over the past two decades (Flegal, 2005) has affected every age group including older adults (Villareal et al., 2005). Because obesity is related to numerous diseases of aging and is also a risk factor for physical disability (Rejeski et al., 2010), there has been an increased interest in weight loss for older adults (Villareal et al., 2006; Rejeski et al., 2011), and an attempt to better understand the etiology of excessive energy intake in this subgroup of the population (Rejeski et al., 2012). In the current study, we examine how an important individual differences variable, the Power of Food Scale (PFS; Lowe et al., 2009), may moderate brain networks of older adults during a procedure in which they were asked to create a multi-sensory image involving the consumption of their favorite food.

The role of visual stimuli in the drive to consume food has captured the interest of investigators for some time (Cornier, 2011). Food choices and cravings, like many other behaviors, are known to be heavily influenced by the visual system (Laska et al., 2007). Several recent studies including a meta-analysis have shown that the visual cortex is active during the processing of food-related visual cues (Cornier et al., 2007; van der Laan et al., 2011). Using a cognitive experimental approach, Tiggemann and colleagues (Tiggemann and Kemps, 2005; Kemps et al., 2008a) have shown that mental imagery is a key component of food cravings; however, to our knowledge no imaging studies have examined how the imagery of food affects the visual cortex or whether such responses may be moderated by important individual difference variables such as the PFS.

The current study involved a controlled laboratory protocol in which older, overweight/obese adults were fed breakfast and restrained from eating for 2.5 h on two separate occasions. Following one period of restraint, participants were fed a liquid meal replacement, BOOST^®^, whereas the other involved the consumption of water. Participants then had an MRI scan following the consumption of either BOOST^®^ or water (NO BOOST^®^) during which they imaged both food and neutral cues. The aims of the current study were to examine how these two different imagery conditions affected cravings and functional connectivity in the visual cortex. We expected cravings to be elevated following imagery of food as compared to neutral cues and hypothesized that persons scoring high on the PFS would have higher cravings to food than those scoring low on this measure. A particularly novel feature of this study is that we compared local connectivity, or local efficiency, within the visual cortex during the actual imagery of both neutral and food cues for participants scoring low and high on the PFS. We hypothesized that participants scoring high on the PFS, as compared to those scoring low, would exhibit higher local efficiency within the visual cortex when imaging food cues; no such difference was expected when imaging neutral cues. We also expected that any group differences in local efficiency would be magnified by the NO BOOST^®^ treatment condition.

## MATERIALS AND METHODS

### PARTICIPANTS

A sample (*n* = 14) of older, overweight/obese (BMI ≥ 28 but ≤40 kg/m^2^) adults was recruited from Forsyth County, NC, USA. All participants were between the ages of 50 and 79 and lived independently. Each participant completed a phone screen, an in-person screening visit, and two 5 h experimental sessions at Wake Forest School of Medicine, receiving a maximum of $225 for completing all three visits to compensate for their time.

### PRESCREENING AND LOST TO FOLLOW-UP

A telephone screen was administered to interested individuals. Our target population was comprised of older, overweight/obese adults falling into two categories: “high PFS” or “low PFS.” These groupings based on the PFS administered during screening are explained in further detail below. Exclusion criteria for the study included: (1) scores on the PFS falling between 2.5 and 3.0, (2) having a BMI lower than 28 or >40kg/m^2^, (3) the presence of a systemic uncontrolled disease or psychiatric illness, (4) a binge eating disorder, (5) high alcoholic intake (more than three drinks per day), (6) the inability to safely undergo magnetic resonance imaging due to claustrophobia or to the presence of implanted magnetic objects/devices, (7) currently undergoing treatment for cancer, (8) active participation in another research study, (9) need for assistance while walking, (10) being unable to read or speak English, or (11) the inability to correct eyesight to at least 20/40 in the MRI scanner to complete the required tasks.

Over 125 people were contacted by phone and 22 attended in-person screening visits. Of these 22, 7 were not eligible for the study due to various reasons including claustrophobia, scoring between 2.5 and 3.0 on the PFS when retested, and being unable to correct for their vision in the scanner. One participant was excluded due to poor quality of brain images during one functional scan that could not be corrected using computer software; thus, the final *n* was 14.

### MEASURES

#### Power of food scale

The PFS assesses the drive to consume highly palatable food in an obesogenic food environment. Higher scores are associated with higher drive. The total score has been shown to have good test-retest reliability (*r* = 0.77), is internally consistent (α = 0.91), and support exists for its construct validity (Cappelleri et al., 2009; Lowe et al., 2009). Three subscale scores can be calculated: food available, food presence, and food tasted. In this study we use the total score due to the high level of internal consistency within the test. To create two groups of participants, high and low, we intentionally eliminated people scoring in the middle of the range 2.5–3.0. Thus we defined our high PFS group (*n* = 7) to be those scoring >3.0 and our low PFS group (*n* = 7) to have scores <2.5.

#### Confidence for controlling eating behavior

Consistent with the work of Bandura (1991), we have developed a four item measure of self-efficacy for eating restraint for favorite foods that is state-based. That is, the measure requires participants to rate their confidence in being able to resist or control eating their favorite food right now, at this moment. The four items are as follows: (1) if available, I could resist eating my favorite foods; (2) at the current time, I feel like I have good control over my appetite; (3) at the moment, I feel as if I could restrain myself from eating foods that I enjoy; and (4) currently, I feel that I could avoid snacking between planned meals. The items are rated on a 10 point scale ranging from 0 “not at all confident” to 10 “very confident,” with the anchor “moderately confident” spanning the values from 4 to 6 and centered at 5. The measure has good internal consistency (Cronbach alpha reliability of 0.90) and is responsive to food restriction (Rejeski et al., 2012).

#### The interview for the diagnosis of eating disorders

The semi-structured interview described by Kutlesic and colleagues (Kutlesic et al., 1998) was employed to exclude any potential participants that might have a binge-eating disorder as defined by the DSM-IV criteria. Dr. Williamson, an investigator involved in the development of the interview for the diagnosis of eating disorders (IDED-IV), provided the training on how to screen for Binge Eating.

### IN-PERSON SCREENING AND ASSESSMENTS

An in-person screening visit was completed to obtain an informed consent, collect biometric data, assess current states of physical activity, and possible dieting, and to screen for binge eating disorders using the IDED-IV. Potential participants also completed the PFS. Eligible individuals were scheduled for two imaging visits 7–10 days apart. If necessary, participants were fitted for MRI-safe corrective lenses to be used in the scanner during reading tasks.

### EXPERIMENTAL PROTOCOL FOR THE SCANNING VISITS

**FIGURE 1:**
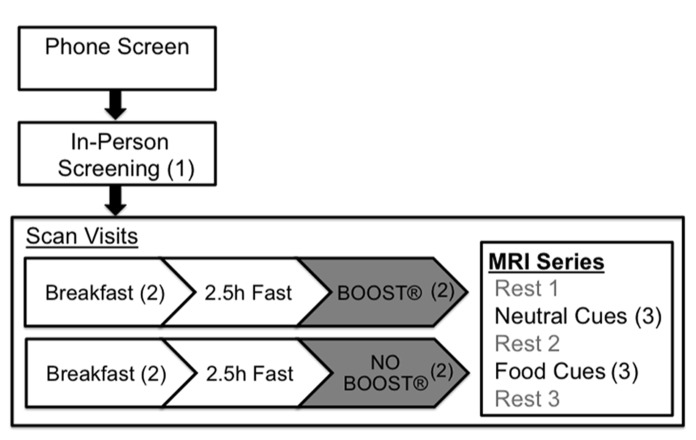
**Visit progression from phone screening to scan visits, indicating points of collection for PFS (1), CCEB_state_ (2), and VAS (3) scores**.

Participants completed two 5 h visits beginning in the early morning around 8:00 a.m. Participants arrived in a fasting state, having not eaten breakfast, or drank anything other than water. During each visit, participants consumed a prepared breakfast containing 350 calories for females and 450 calories for males. The meals were designed by a staff nutritionist to provide a heart healthy balance of macronutrients containing approximately 25% fat, 15% protein, and 60% carbohydrates. Participants were allowed to choose macronutrients from a menu. Following the consumption of at least 75% of their breakfast, participants completed the Confidence for controlling eating behavior (CCEB_state_). The participants then fasted for 2.5 h under the supervision of research staff (see **Figure 1** for overview of study design).

Approximately 45 min before the imaging procedure, participants were asked to identify and rate the pleasantness of two favorite foods and then choose the food they craved most that day. These favorite foods were used as food cue words during neuroimaging. The research staff then administered the MRI safety form and led the participant in a practice session of the tasks to be completed during the fMRI. Following the practice session, about 30 min before the scan, participants either consumed a can of BOOST^®^ (short term energy surfeit containing 240 calories) or an equivalent volume of water (termed the NO BOOST^®^ condition). They then completed a second round of the CCEB_state_. The order of assignment of BOOST^®^ and NO BOOST^®^ to each participant was randomized.

### NON-FOOD AND FOOD- CUE SCANNING TASKS

Participants wore goggles in the scanner that were directly interfaced with a computer screen. The tasks they performed involved the visualization of words that were presented on the computer screen for approximately 25 s each. There were two separate task sessions: one involving neutral cues and one with food cues. During the food-cue session, the words shown were each participant’s favorite foods; words chosen for the neutral cue condition were selected using the International Affect Picture System (Lang et al., 2005). We matched each food word with a neutral word beginning with the same letter, having approximately the same total amount of letters, and having a neutral valence (4.5–5.5). For both tasks, the words were presented in a random order with the restriction that each of the four words was presented at least once in each session. Each task session was both preceded and followed by a rest session so that the sequence was as follows: rest 1, neutral cues, rest 2, food cues, and rest 3 (see **Figure 1**). All sessions were administered in continuous 5 min blocks. For analytic purposes, this paper focuses on data collected during the food and neutral cue imagery conditions.

The instructions for the visualization task sessions were as follows: “During the task you will see words on the screen in front of you. Some of these words describe your favorite foods and others are non-food related. Each time a word appears, I want you to think about that word and what it represents. So, for example, if the words ‘baked potato’ appeared, imagine the ingredients that you like to put on the potato, see the steam coming from it, think about how it smells, its texture, and how it would taste. *I want you to try and use as many of your senses as possible to come up with the best image you can.* Hold on to that image for the entire time that the word is on the screen. Now, I want you to do the same thing for the non-food words. So, if the word ‘desk’ appears, where is it? How many drawers does it have? Is the wood dark or light? Is it rough or smooth? Once again, hold on to that image for the entire time it is on the screen. There will be a cross on the screen at the beginning and end of each visualization session and for the entire duration of the rest sessions, during those times just focus on the cross and do not think about anything in particular. For each visualization task you will be asked to provide ratings of (1) your hunger, (2) craving for your favorite food, and (3) how vivid the image was. You will see your response to these scales using computer images that appear in your goggles.” Immediately following each visualization block, neutral cues, and food cues, participants provided ratings for their hunger, level of craving, and vividness of the images using visual analog scales (VAS) ranging from 0 (“not at all”) to 100 (“extreme”/”very well”).

### SCANNING PROTOCOL

All scans were performed on a 1.5 GTE scanner using an eight channel neurovascular head coil (GE Medical Systems, Milwaukee, WI, USA). Functional images for the network analyzes measured changes in the T2* relaxation rate that accompany changes in blood oxygenation. The T2* signal is sensitive to changes in blood oxygen content. As brain activity changes, the oxygen content of the blood in the same area also changes. Thus, the T2* signal is an indirect measure of changes in neural activity (Ogawa et al., 1990). Functional imaging was performed using multi-slice gradient EPI (TR = 2000 ms; TE = 40 ms; field of view = 24 cm (frequency) × 15 cm (phase); matrix size = 3.75 mm × 3.75 mm × 5 mm).

### STATISTICAL ANALYSES OF SELF-REPORT DATA

We used mixed model ANCOVAs to test the effect of the BOOST^®^ manipulation and the PFS on the CCEB_state_. In these analyses, we controlled for the post-breakfast feeding assessment as well as the random subject effect. The outcomes of interest were the responses taken just prior to conducting the fMRI scans. We also tested for interactions between the BOOST^®^ manipulation and the PFS. To examine VAS responses during the presentation of neutral and food cues during the scanning procedure, we employed 2 (PFS category: high vs. low) × 2 (cue type: neutral vs. food) general linear models with a repeated measures for cue type. All tests were conducted using a 0.05 level of significance except for interaction effects which were evaluated at the *p* < 0.10 level (Snedecor and Cochran, 1967).

### IMAGING PROCESSING AND NETWORK ANALYSES

In preparation for generating brain networks, all scanning images were realigned and normalized to standard space using FSL software (Smith et al., 2004). The time courses were extracted for each voxel in gray matter based on the Automated Anatomical Labeling atlas (Tzourio-Mazoyer et al., 2002) and band-pass filtered to remove signals outside the 0.009–0.08 Hz range (Biswal et al., 1995). To account for physiological noise, mean white matter, cerebral spinal fluid (CSF), global signal, and motion correction parameters were regressed from the filtered time series. A correlation matrix was then created by computing Pearson correlations between the remaining signals in all possible pairs of voxels (~21,000 voxels). This produced a ~21,000 × 21,000 matrix which each cell (*ij*) representing the correlation coefficient between nodes *i* and *j*. A threshold was then applied to the correlation matrix and all cells that surpassed this threshold were assigned a value of 1 while remaining cells were assigned a value of 0. The threshold was defined so that the relationship between the number of nodes and average number of connections at each node was consistent across subjects to produce an adjacency matrix. Specifically, the relationship *S* = log (*N*)/log(*K*) was the same across subjects as described above (Hayasaka and Laurienti, 2010). The threshold *S* = 2.5 was used for this paper. All remaining analyses were completed using the binary adjacency matrix.

To evaluate network organization, a metric which assesses the role of each node in sharing information with neighboring nodes, specifically, local efficiency was used (Latora and Marchiori, 2001). This metric quantifies the amount of interconnectivity between the neighbors of a specific node (*i*). In brief, for each node *i*, the neighbors are identified as those with shared network connections. The sub-network (G**_i_) containing all the neighbors of node *i* is constructed. Then the shortest distance or path length (d**_jkj_), between each of the node pairs in G**_i_ is determined. The path length is the fewest number of edges one must traverse to get from node *j* to node *k*. Local efficiency of node *i* is calculated as:

$$E_{loc}=\frac{1}{N\,\left(N-1\right)}\,\sum_{j\neq k\in G_{i}}\frac{1}{d_{jk}}$$

Thus, *E*_loc_ is the average shortest path length between all of the nodes in G**_i_ that are neighbors of node *i*. *E*_loc_ ranges from 0 to 1 and determines how efficient the communication is between the neighbors of a node *i* when *i* is removed. A node with neighbors that are fully interconnected would have a local efficiency of 1; a node with neighbors that share no connections would have a local efficiency of 0. Spatial consistency across subjects for nodes containing the highest values of local efficiency was then determined by identifying the top 20% of nodes and then overlapping the location of those nodes across study subjects. This provides a clearer picture of the consistency of the spatial location of high efficiency nodes across subjects.

## RESULTS

Participants had a mean (SD) age of 71.35 (4.92) years with a Body Mass Index of 30.43 (2.09). Twelve were White and two were Black with the leading comorbidities including arthritis (*n* = 8) and hypertension (*n* = 4); these health conditions were followed by cancer (*n* = 2) and cardiovascular disease (*n* = 1). The PFS groups did not differ on BMI or age (*p* > 0.05); mean (SD) BMI, and age for low and high groups were 30.5 (2.1) vs. 30.4 (2.3) and 70.3 (4.1) vs. 72.4 (5.7), respectively. However, there was an imbalance for sex with five men and two women in the low PFS group and five women and two men in the high PFS group. This imbalance was not found to influence interpretation of the study outcomes. The efficacy of the BOOST^®^ manipulation was supported by data showing that ratings of hunger as assessed by Cepeda-Benito and colleagues measure (Cepeda-Benito et al., 2000) were higher on the day that participants consumed water prior to the scanning procedure, mean (SE) = 9.00 (0.67), as compared to the day they received BOOST^®^, mean (SE) = 6.78 (0.75); *t*_13_ = 2.14 (13), *p* = 0.05.

### Perceived control

**FIGURE 2:**
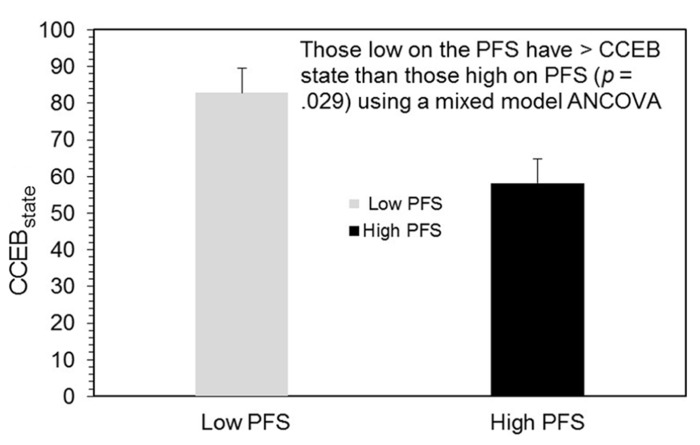
**CCEB state scores by PFS grouping**.

As discussed previously, a mixed-model ANCOVA was employed to examine how participants’ self-confidence for controlled eating behavior (CCEBstate) was influenced by the two treatment conditions (BOOST^®^ or NO BOOST^®^) and whether this effect was moderated by scores on the PFS. This analysis yielded a significant main effect for the PFS (*F*_1,11_= 2.51, *p* = 0.029; see **Figure 2**) indicating that participants with low PFS scores had higher CCEB_state_ scores those with high PFS scores [mean (SE) = 82.77 (6.65) and 58.19 (6.65), respectively]. There were no other statistically significant effects in this model.

### VAS responses to food and neutral cues

**FIGURE 3:**
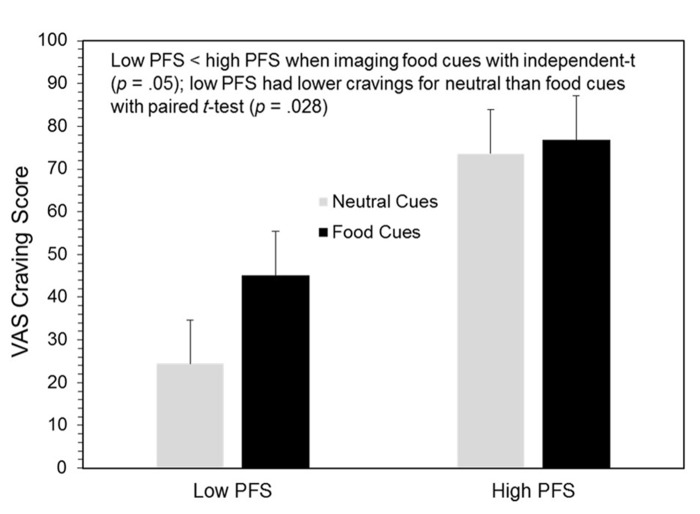
**VAS craving scores by cue types and low vs. high power of food scale (PFS) scores**.

A general linear model for repeated measures on the VAS craving measure yielded significant main effects for cue type (*F*_1,12_= 6.54, *p* = 0.025) and the PFS (*F*_1,12_= 7.67, *p* = 0.017), as well as a significant interaction between these effects (*F*_1,12_= 3.52, *p* = 0.082.). As hypothesized, craving was lower in the neutral, mean (SE) = 49.01 (8.03), than food cue condition, 60.95 (7.27); participants high on the PFS reported higher cravings, 75.19 (10.32), than those scoring low on the PFS, 34.77 (10.32). An interesting finding was that cravings for participants high on the PFS were quite high and no different in either the neutral or food cue conditions, 73.60 (11.36) vs. 76.79 (10.28), while this was not true for those low on the PFS who had very low cravings following the neutral cue exposure, 24.43 (11.30), and moderate cravings following food imagery, 45.10 (10.28); *p* = 0.028 (see **Figure 3**). As hypothesized, within the food imagery condition, those scoring low on the PFS had lower cravings than those scoring high on the PFS, *p* = 0.05. The only other significant effect for the VAS scales was that hunger was rated higher following imagery of food, 60.98 (9.10), than neutral cues, 50.21 (9.70); *F*_1,12_= 6.66, *p* = 0.024. Although vividness ratings appeared to be higher when participants imagined food, 84.42 (4.28), as compared to neutral cues, 74.30 (6.99), this difference was not statistically significant; *p* = 0.18. Also, people scoring low and high on the PFS had vividness ratings that were essentially identical, 85.86 (6.05) vs. 83.00 (6.05), respectively.

### Network analyses during imagery

**FIGURE 4:**
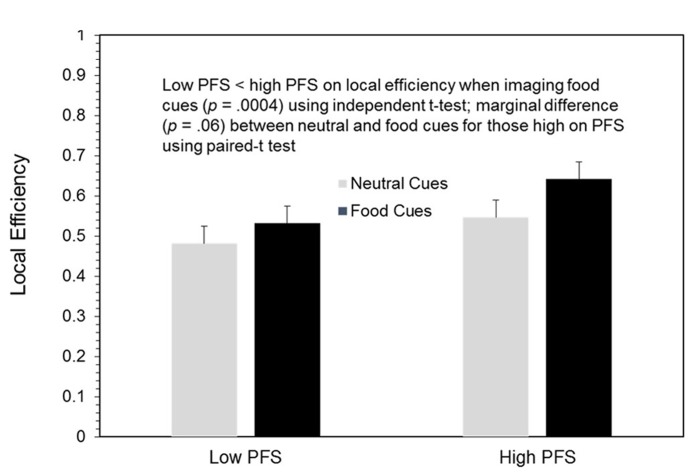
**Sagittal and axial slices highlighting the visual cortex (red circles) show nodes with high local efficiency (top 20%).** Color bar represents percentage of participants within each group for whom local efficiency was high in each area.

**FIGURE 5:**
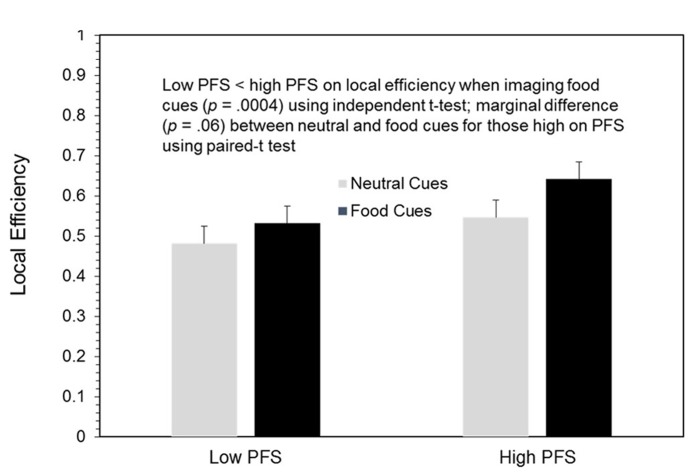
**Local efficiency scores by cue types and low vs. high power of food scale (PFS) scores**.

Because there were no significant differences between the BOOST^®^ and NO BOOST^®^ treatment conditions in the network analyses, we focus our presentation in **Figures 4** and **5** on local efficiency in the visual cortex for low and high PFS participants during the processing of both neutral and food cues using data from the NO BOOST^®^ treatment condition. As hypothesized and shown in **Figure 4**, local efficiency in the visual cortex (shown in the red circle) during imagery of food was greater for persons with high than low PFS scores. Also, among the high PFS group, local efficiency appeared to be higher for the food than neutral cues. **Figure 5** provides the same data for local efficiency in a bar chart. An ROI analysis was performed to statistically compare the connectivity within the visual cortices of persons low and high on the PFS using two 10 mm spheres (MNI coordinates: 22, -92, 4 and -12, -92, 4). Results revealed that the high PFS group had significantly higher local efficiency in the visual cortex than the low PFS groups during visualization of food cues, means (SEs) = 0.642 (0.023) and 0.532 (0.022), respectively (*p* = 0.004). There was a trend for the local efficiency of participants high on the PFS to be higher in the food than neutral cue condition, *p* = 0.06.

## DISCUSSION

The topic of food craving has become increasingly popular in the scientific community because of the academic interest in the concept of *desire* (Kavanagh et al., 2005) and the health challenges posed by both obesity (Abelson and Kennedy, 2004) and disordered eating (Kemps and Tiggemann, 2010). Using a cognitive experimental paradigm, Kemps and Tiggemann (2010) have provided evidence that imagery is a prime driver of food cravings. They have shown that the experimental induction of food craving via imagery slows reaction time and reduces working memory (Kemps et al., 2008b). Furthermore, when they had participants engage in a visual noise task, a procedure that competes for the limited processing capacity of the visual system, they observed reductions in food cravings among prescribed weight loss dieters (Kemps et al., 2008a). Also worth noting is that they have found increased vividness ratings when people created images of food as compared to neutral objects.

The current study was designed to build upon this body of literature. First, using visual analog scales during an fMRI scan, we observed marked increases in food cravings when older, overweight/obese adults created images of favorite foods in their minds as opposed to creating an image of neutral words. This finding was consistent with the higher hunger ratings that participants gave following the imagery of food as compared to neutral words. A fascinating pattern in the data was that persons scoring high on the PFS reported high cravings irrespective of the imagery condition, whereas those low on the PFS had lower cravings following the neutral than food imagery manipulation; moreover, even in the food condition, low PFS participants had ratings below those scoring high on the PFS. In this regard, it is worth noting that, prior to the scanning process, persons scoring high on the PFS reported less confidence in being able to control consuming favorite foods than did those scoring low on the PFS. Although ratings for vividness of imagery were essentially identical for those scoring either low or high on the PFS, and the difference between cue conditions did not reach statistical significance, Kemps and Tiggemann (2010) reported that vividness ratings were higher for food than neutral imagery. We suspect that this lack of agreement is most likely due to our limited power given the sample size limitations imposed by the cost of scanning.

A unique feature of the current study was our ability to examine brain networks during the imaging of both neutral and food cues. Based on our previous work (Rejeski et al., 2012), we expected that the BOOST^®^ manipulation would accentuate differences in the visual cortex between the neutral and food cue trials, however, this did not occur. Rather, irrespective of the BOOST^®^ manipulation, participants who scored high on the PFS had greater connectivity in the visual cortex during the imaging of food cues than those scoring low on the PFS. Thus, the active imagery of desired foods seemed to have overpowered the BOOST^®^ manipulation when evaluating connectivity in the visual cortex.

We find the lack of correspondence between patterns in the VAS craving data and connectivity in the visual cortex to be intriguing. First, when asked to rate craving for favorite foods, it would appear that *people scoring high on the PFS are more trait- than state-like*; they have a high drive to consume food that does not dissipate with focusing their attention on neutral words. In this regard, it would be interesting to see whether visual noise, as reported by Kemps et al. (2008a), is effective in altering food cravings for this subgroup. The fact that persons high and low on the PFS do not differ when asked to rate the vividness of their imagery, suggests that it is something other than the clarity of the image that is responsible for the connectivity differences in the visual cortex between PFS groups in the food imagery condition. Second, while visual imagery and concurrent activity in the visual cortex may contribute to self-reported cravings for desired food and to dysfunctional consumption found with obesity and disordered eating (Kemps and Tiggemann, 2010), there are clearly important individual differences that deserve study within the realm of network science (Paolini et al., 2012; Rejeski et al., 2012).

This current study is not without limitations. First, the target sample was small and restricted to an older, overweight/obese population that was not currently dieting. Also, we did not have a normal weight control group, meaning that these results may be limited to older adults that are obese. This is an area that warrants attention in the future. Second, cravings were reported immediately following rather than during the actual imagery condition and this might explain the lack of correspondence between responses to the VAS measures and connectivity in the visual cortex. However, if this was the case, one would not have expected the differences that we did observe with the VAS craving scale. And third, the experimental paradigm did not allow us to evaluate whether differences in the visual cortex are predictive of differences in actual consumption of food. The answer to this question awaits further study.

In summary, the patterns observed in brain networks within this experimental paradigm reinforce the powerful role that food imagery has on the visual cortex of people who feel overpowered by the obesogenic environment common to Western Society. Also the self-report of craving for older adults high on the PFS seems to be trait-like; that is, persistent following both neutral and food imagery. An important question is whether brain signatures in response to a brief experimental paradigm such as this are predictive of failure at self-regulating the intake of calories and unhealthy macronutrients. This is an extremely important area for future research and one that we are pursuing within the context of a long-term weight loss trial. In addition, the markedly higher VAS ratings for craving during the imagery manipulations on the part of participants scoring high on the PFS suggests that this subgroup may have a particularly difficult time adhering to reduced caloric intake prescribed during weight loss interventions and that focused attention may be needed to deal with the management of cravings in people who score high on the PFS.

## Conflict of Interest Statement

The authors declare that the research was conducted in the absence of any commercial or financial relationships that could be construed as a potential conflict of interest.

## AUTHOR CONTRIBUTIONS

Jessica Bullins running participants, processing scans, writing first draft, Paul J. Laurienti editing of drafts, reviewing and interpretation of scans, Ashley R. Morgan running participants and processing the scans, James Norris statistical analysis and interpretation, Brielle M. Paolini running participants, assisting with processing and interpretation of scans, W. Jack Rejeski provided conceptual foundation for paper, writing of drafts and interpretation of data.
